# Non-invasive and invasive measurement of skeletal muscular oxygenation during isolated limb perfusion

**DOI:** 10.1177/02676591221093201

**Published:** 2022-05-16

**Authors:** Anna Corderfeldt Keiller, Anna Holmén, Christoffer Hansson, Sven-Erik Ricksten, Gudrun Bragadottir, Roger Olofsson Bagge

**Affiliations:** 1 Department of Cardiothoracic Surgery, Sahlgrenska University Hospital, Gothenburg, Sweden; 2 Department of Anesthesiology and Intensive Care Medicine, Sahlgrenska Academy, University of Gothenburg, Sahlgrenska University Hospital, Gothenburg, Sweden; 3 Department of Surgery, Institute of Clinical Sciences, Sahlgrenska Academy at the University of Gothenburg, Gothenburg, Sweden; 4 Department of Surgery, Sahlgrenska University Hospital, Gothenburg, Sweden; 5 Wallenberg Centre for Molecular and Translational Medicine, University of Gothenburg, Gothenburg, Sweden

**Keywords:** Isolated limb perfusion, extracorporeal circulation, oxygen delivery, oxygen extraction, near infrared spectroscopy

## Abstract

**Background:**

Isolated limb perfusion (ILP) is a regional surgical treatment for localized metastatic disease. High doses of chemotherapeutic agents are administered within an extracorporeal circulated isolated extremity, treating the metastasis, while systemic toxicity is avoided. To our knowledge, indexed oxygen supply/demand relationship during ILP has not previously been described. Our aim was to measure and describe oxygen metabolism, specifically oxygen delivery, consumption, and extraction, in an isolated leg/arm during ILP. Also investigate whether invasive oxygenation measurement during ILP correlates and can be used interchangeable with the non-invasive method, near infrared spectroscopy (NIRS).

**Methods:**

Data from 40 patients scheduled for ILP were included. At six time points blood samples were drawn during the procedure. DO2, VO2, and O2ER were calculated according to standard formulas. NIRS and hemodynamics were recorded every 10 min.

**Results:**

For all observations, the mean of DO2 was 190±59 ml/min/m2, VO2 was 35±8 ml/min/m2, and O2ER was 21±8%. VO2 was significantly higher in legs compared to arms (38±8 vs. 29±7 ml/min/m2, p=0.02). Repeated measures showed a significant decrease in DO2 in legs (209±65 to 180±66 ml/min/m2, p=<0.01) and in arms (252±72 to 150±57 ml/min/m2, p=<0.01). Significant increase in O2ER in arms was also found (p=0.03). Significant correlation was detected between NIRS and venous extremity oxygen saturation (SveO2) (rrm=0.568, p=<. 001, 95% CI 0.397–0.701). When comparing SveO2 and NIRS using a Bland–Altman analysis, the mean difference (bias) was 8.26±13.03 (p=<. 001) and the limit of agreement was − 17.28–33.09, with an error of 32.5%.

**Conclusion:**

DO2 above 170 ml/min/m2 during ILP kept O2ER below 30% for all observations. NIRS correlates significant to SveO2; however, the two methods do not agree sufficiently to work interchangeable. Clinical Trial Registration URL: https://www.clinicaltrials.gov. Unique identifier: NCT04460053 and NCT03073304.

## Introduction

Isolated limb perfusion (ILP) is a regional treatment for malignancies of the extremity.^1^ A heart and lung machine (HLM) is connected to a surgically isolated extremity, which enables oxygenation, perfusion, hyperthermia, and delivery of high doses of chemotherapeutic agents within the extremity while systemic toxicity is avoided.^2,3^ The drug is perfused trough the limb during 60–90 min. Before re-establishing the system circulation, the extremity is irrigated with crystalloid to remove the chemotherapeutic agents.^4^ The method has been refined over time, and the current overall response rate for patients with in-transit metastasis of melanoma is ranging between 65 and 100%, with a complete response rate between 25 and 76%.^4,5^

The primary goal of the cardio-respiratory system is to deliver adequate amount of oxygen to the tissues to meet their metabolic requirements. The adequacy of tissue oxygenation is determined by the balance between the rate of oxygen transport to the tissues (DO_2_) and the rate at which the oxygen is used by the tissues (VO_2_), also known as the oxygen supply/demand relationship.^6^

During extracorporeal circulation (ECC), DO_2_ is foremost dependent on blood flow, oxygen saturation, and hemoglobin concentration. If either of these factors decreases, the oxygen extraction ratio (O_2_ER) in organs increases to meet metabolic needs. In case DO_2_ falls below critical levels and O_2_ER exceeds 40%–50%, VO_2_ gradually decrease in proportion to the decrease in DO_2_ and a pathologic supply dependency arises as evidence of O_2_ debt. Cells enter an anaerobic metabolism phase and an increase in lactate levels occurs.^7–9^

Substantial amount of research has been conducted regarding the nadir level of oxygen delivery. In an anesthetized patient (34–36°C) undergoing open heart surgery on cardiopulmonary bypass (CPB), the sufficient amount of DO_2_ appears to be 265–300 ml/min/m^2,8,10–17^ and a safe upper limit for O_2_ER seems to be 39%.^18^ The perfusion flowrate commonly used during cardiac surgery for whole body perfusion is 2.2–2.5 L/min/m^2^, which approximates the patients’ cardiac index based on patient’s body surface area (BSA).^15,19^ A newer form of flowrate setting is goal-directed perfusion, where DO_2_ is continuously measured during CPB, with the aim to stay above nadir values of DO_2_ through the perfusion.^11,13,16,17^ Effective DO_2_ during CPB can continuously be monitored by VO_2_ and O_2_ER.^20^ During ILP only one extremity is perfused, compared to a total body perfusion during open heart surgery, where all vital organs must be optimally perfused. Approximately 25% of the cardiac output (generally 5–6 L/min at-rest)^21^ is destined to skeletal muscles.^22^ According to the Wallace Rule of Nines, a leg constitutes approximately 18% of the BSA and an arm approximately 9% of total BSA.^23^ Perfusion of one leg would then require a cardiac output of approximately 1.0 L/min and 0.5 L/min for an arm.

In 1977, Jöbsis^24^ described for the first time the potential in using infrared (IR) spectroscopy as a real-time, non-invasive, continuously measuring tool for tissue oxygen saturation (rSO_2_).^24^ Near-infrared spectroscopy (NIRS) devices measure mean tissue oxygen saturation reflecting hemoglobin saturation in a mixture of venous, capillary, and arterial blood. Average tissue hemoglobin is distributed in a biologic variation in an arterial/venous ratio of approximately 25:75, which is similar during normoxia, hypoxia, and hypocapnia.^25,26^ Thus, the NIRS signal is to a great extent reflecting tissue venous oxygen saturation. Research has demonstrated that NIRS, beside cerebral oxygenation monitoring, may be used as a non-invasive way of measuring muscular oxygenation, that is, the muscular oxygen supply/demand relationship, detecting skeletal muscle ischemia in the human leg as well as other peripheral tissues.^27–31^

The overall aim of the study was to measure and describe oxygen metabolism, specifically oxygen delivery, consumption, and extraction, in an isolated leg/arm during ILP. Our hypothesis was that no changes in oxygen supply/demand relationship will occur over time in the ECC-perfused extremity during ILP. Furthermore, we also examined if there was a correlation and agreement between non-invasive NIRS and invasive muscular oxygenation measurement during ILP.

## Patients and methods

### Patients

Forty patients derived from two prosepective clinical studies of ILP (Corderfeldt et al.32 and ClinicalTrials.gov, identifier: NCT04460053) was included after written informed consent was obtained. The exclusion criteria were leakage >0 ml blood from the system circulation to the isolated extremity, and a leakage of >10% chemotherapeutic agents from the extremity to the system circulation. 19 patients were excluded due to exceeding the waste limitation of 0 mL and one patient was excluded due to leakage of chemotherapeutic agents to the patient of >10%. The final analysis contained 20 patients. Ethical approval was issued by the Swedish ethical board (Dnr. 1145-16 and Dnr. 2019-01046). The study was registered in ClinicalTrials.gov (Identifier: NCT04460053 and NCT03073304).

### Clinical management

In all patients, anesthesia was induced with propofol (1.5–2.5 mg/kg), fentanyl (1.0–3 μg/kg), rocuronium (0.6 mg/kg), and maintained with sevoflurane. The ECC circuit was primed with 500 mL of Ringer-Acetate (Fresenius Kabi AB, Uppsala, Sweden), 100 mL Tribonat (Fresenius Kabi AB), 100 mL Albumin 200 g/L (Baxalta, Illinois City, USA), and 2500 IU heparin (LEO Pharma, Ballerup, Denmark) for leg perfusion. The priming solution for arm perfusion was the same as for the leg except for 1 unit of packed red cells (250 mL) which was added together with only 250 mL of Ringer-Acetate. The difference in prime regime between extremities is due to potential hemodilution anemia in arms related to large prime volume/low surface area in arms compared to legs. The ILP technique, ECC assembling, leakage monitoring, and temperature measurements were performed according to clinical routine as described by Corderfeldt et al.^32^

### Measurements

Blood was sampled at six different time points during the procedure:

1. After induction (arterial cannula).
2. Pre-perfusion (only O_2_ER and SveO_2_) (arterial cannula and punction from femoral vein).
3. ECC start (arterial- and venous blood sampled from ECC).
4. After chemotherapeutic infusion (arterial- and venous blood sampled from ECC).
5. At the end of perfusion, before rinsing (arterial- and venous blood sampled from ECC).
6. 10 min after release of the isolation when system circulation is re-established (arterial cannula and punction from femoral vein).

An NIRS monitoring, INVOS^®^ 5100c Cerebral/Somatic Oximetry Adult Sensor (Medtronic, Minneapolis, USA) was used for measuring rSO_2_. The sensors were placed on the leg/arm (bilateral) on the tibialis-/brachioradialis muscle. NIRS and hemodynamics were recorded every 10 min.

### Oxygen calculations

The oxygen content of the arterial blood (CaO_2_) and venous blood (CvO2), DO_2_, VO_2_, O_2_ER, and pump flow (Q) were calculated according to standard formulas.^10,12,14,33^

CaO_2_ = (1.36 × Hb (g/dL)) x (SaO_2_ (%) x 0.01) + (0.023 × PaO_2_ (kPa))
CvO_2_ = (1.36 × Hb (g/dL)) x (SvO_2_ (%) x 0.01) + (0.023 × PaO_2_ (kPa))
DO_2_ indexed = Q (L/min) x CaO_2_/BSA^extremity^
VO_2_ indexed = Q x (CaO_2_-CvO_2_)/BSA^extremity^
O_2_ER = VO_2_/DO_2_

To be able to compare DO_2_, VO_2_, and O_2_ER between patients with various limb sizes, we indexed these parameters to extremity surface area instead of total body surface area. The patient’s limb circumference was measured every 5 cm, the mean circumference was then multiplied with the length of the extremity. The individually adjusted chemotherapy doses are also based on these measurements and are performed routinely prior ILP.

### Statistical evaluation

Data are presented as mean ± standard deviation (SD) unless otherwise stated. Variables were tested for normality with Shapiro–Wilk test. Treatment characteristics on ECC were analyzed with independent-samples T Test. One-way repeated measures ANOVA was used to detect changes in mean over time during perfusion and a paired-sample T test for significant ANOVA parameters. A probability level (*p*-value) of less than 0.05 was considered statistically significant. To calculate the relationship between several, repeated measurement points for NIRS and invasive muscular oxygenation (extremity venous oxygen saturation [SveO_2_], partial pressure of oxygen [PaO_2_], and arterial oxygen saturation [SaO_2_]) in one individual, we used repeated-measures correlation analyses.^34^ The repeated-measures correlation coefficients (r_rm_), representing the strength of the linear association between the variables, were calculated. The agreement between two methods was assessed according to Bland and Altman.^35,36^ The mean difference between the methods (bias) and the error (double standard deviation of the difference divided by the mean of the measurements from the two methods) and the limits of agreement (mean difference ±2 standard deviations) were calculated. The differences between the two methods are normally distributed (Shapiro–Wilk, *p*=0.33). A priori we defined an acceptable between-method error to be 30% or less according to Critchley and Critchley and Odor et al.^37,38^ Data was analyzed using SPSS version 27 (IBM, Armonk, NY) and R stats version 3.6.3 and rmcorr version 0.4.3 (Vienna, Austria).

## RESULTS

20 patients were included in the final analysis, 15 males (75%) and 5 females (25%). The most common diagnosis was melanoma (75%). 14 patients underwent ILP of a leg and six patients underwent ILP of an arm (Table 1).

**Table 1.** Baseline patient characteristics for total observations, leg, and arm.

^Demographic and health characteristic^	^Leg and Arm (n=20)^	^Leg (n=14)^	^Arm (n=6)^
^Gender^			
^Male, n (%)^	^15 (75%)^	^10 (71%)^	^5 (83%)^
^Female, n (%)^	^5 (25%)^	^4 (29%)^	^1 (17%)^
^Body constitution^			
^Median age, years (range)^	^74 (22–83)^	^74 (29–81)^	^67 (22–83)^
^Height, cm^	^175 ± 11^	^174 ± 12^	^177 ± 10^
^Weight, kg^	^80 ± 17^	^83 ± 15^	^73 ± 20^
^Total body surface area, m2^	^1.98 ± 0.26^	^2.02 ± 0.27^	^1.90 ± 0.26^
^Extremity surface area, m2^	^0.28 ± 0.10^	^0.33 ± 0.50^	^0.14 ± 0.21^
^Diagnosis^			
^Melanoma, n (%)^	^15 (75%)^	^11 (79%)^	^4 (67%)^
^Sarcoma, n (%)^	^4 (20%)^	^2 (14%)^	^2 (33%)^
^Neuroendocrine carcinoma, n (%)^	^1 (5%)^	^1 (7%)^	^0 (0%)^
^Preoperative blood work^			
^Hemoglobin, g/L^	^135 ± 17^	^138 ± 16^	^126 ± 18^
^Hematocrit, %^	^39 ± 6^	^40 ± 5^	^37 ± 6^
^Creatinine, μmol/L^	^81 ± 12^	^79 ± 7^	^83 ± 13^
^Potassium, mmol/L^	^4.1 ± 0.4^	^4.2 ± 0.4^	^3.9 ± 0.4^
^Lactate, mmol/L^	^1.04 ± 0.2^	^1.04 ± 0.2^	^1.02 ± 0.3^
^Blood volume in extremity, mL^	^498 ± 172^	^587 ± 109^	^289 ± 83^
^Oxygen parameters before isolation^			
^PaO2, kPa^	^26± 21^	^22 ± 14^	^35 ± 31^
^SaO2, %^	^98 ± 1^	^98 ± 2^	^98 ± 1^
^SveO2 in femoral/brachial vein, %^	^76 ± 14^	^71 ± 13^	^88 ± 6^
^Oxygen extraction, %^	^25 ± 14^	^30 ± 14^	^14 ± 7^
^NIRS-treated extremity, %^	^69 ± 13^	^65 ± 13^	^77 ± 10^
^NIRS not treated extremity, %^	^67 ± 13^	^66 ± 14^	^70 ± 11^
^Comorbidity (%)^			
^No-comorbidity, n (%)^	^6 (30%)^	^4 (29%)^	^2 (33%)^
^Diabetes, n (%)^	^5 (25%)^	^4 (29%)^	^1 (17%)^
^Hypertension, n (%)^	^11 (55%)^	^8 (57%)^	^3 (50%)^
^Hyperlipidemia, n (%)^	^2 (10%)^	^2 (14%)^	^0 (0%)^
^Heart failure, n (%)^	^2 (10%)^	^2 (14%)^	^0 (0%)^
^Other comorbidity, n (%)^	^6 (30%)^	^5 (36%)^	^1 (17%)^

Mean and standard deviation, median, and range or numbers (%). Partial pressure of oxygen (PaO2), Oxygen saturation (SaO2), Venous extremity oxygen saturation (SveO2), and Near infrared spectroscopy (NIRS). Other comorbidity: Ulcerative colitis, Crohn’s disease, rheumatoid arthritis, stroke, and hyperthyroidism.

### Oxygen measurement during ECC

The mean ECC time was 101±9 min, with a mean pump flow of 0.53±0.2 L/min (0.64±0.12 L/min for legs and 0.28±0.07 L/min for arms, *p*=<. 001). The mean SaO_2_ was 99±1%, PaO_2_ was 32±3 kPa, and hemoglobin was 68±15 g/L on ECC. This resulted in a mean DO_2_ of 190±59 ml/min/m^2^, VO_2_ of 35±8 ml/min/m^2^, and O_2_ER of 21±8% for all observations. There was a significant difference in VO_2_ between legs and arms (38±8 vs 29±7 ml/min/m^2^, *p*=. 02) (Table 2).

**Table 2.** Treatment characteristics for total observations, leg, and arm.

Treatment characteristics	Leg and Arm (*n*=20)	Leg (*n*=14)	Arm (*n*=6)	*p*-Value
Time on ECC (min)	101 ± 9	102 ± 8	100 ± 10	0.67
Pump flow (L/min)	0.53 ± 0.2	0.64 ± 0.12	0.28 ± 0.07	<0.001***
FiO^2^ (%)	0.46 ± 0.04	0.47 ± 0.03	0.44 ± 0.05	0.11
Sweep gas flow (L/min)	0.47 ± 0.04	0.47 ± 0.04	0.47 ± 0.04	0.84
Blood work in limb during ECC				
Hemoglobin (g/L)	68 ± 15	67 ± 16	70 ± 13	0.71
Hematocrit (%)	21 ± 5	20 ± 6	21 ± 3	0.75
Lactate (mmol/L)	3.0 ± 1.3	2.5 ± 1.1	4.3 ± 0.8	<0.01**
Oxygen parameters in limb during ECC				
SaO_2_ (%)	99 ± 1	99 ± 1	99 ± 1	0.15
PaO_2_ (kPa)	32 ± 3	33 ± 2	30 ± 4	0.05
SveO2 (femoral/brachial vein) (%)	85 ± 8	83 ± 8	89 ± 4	0.17
DO_2_ (ml/min/m^2^)	190 ± 59	189 ± 62	192 ± 58	0.93
VO_2_ (ml/min/m^2^)	35 ± 8	38 ± 8	29 ± 7	0.02 *
O_2_ER (%)	21 ± 8	23 ± 8	16 ± 5	0.81
NIRS-treated extremity (%)	76 ± 11	73 ± 11	84 ± 9	0.06
NIRS not treated extremity (%)	69 ± 12	68 ± 13	73 ± 8	0.40
Toxicity 1 month follow-up				
Wieberdink (scale, 1–5)	2.35 ± 0.20	2.57 ± 0.94	1.83 ± 0.41	0.08

Mean and standard deviation. Fraction of inspired oxygen (FiO_2_), oxygen saturation (SaO_2_), partial pressure of oxygen (PaO_2_), venous extremity oxygen saturation (SveO_2_), oxygen delivery (DO_2_), oxygen consumption (VO_2_), oxygen extraction (O_2_ER), AND Near infrared spectroscopy (NIRS). Independent-samples T test. *p*<0.05*, *p*<0.01**, *p*<0.001***.

**Figure 1.**
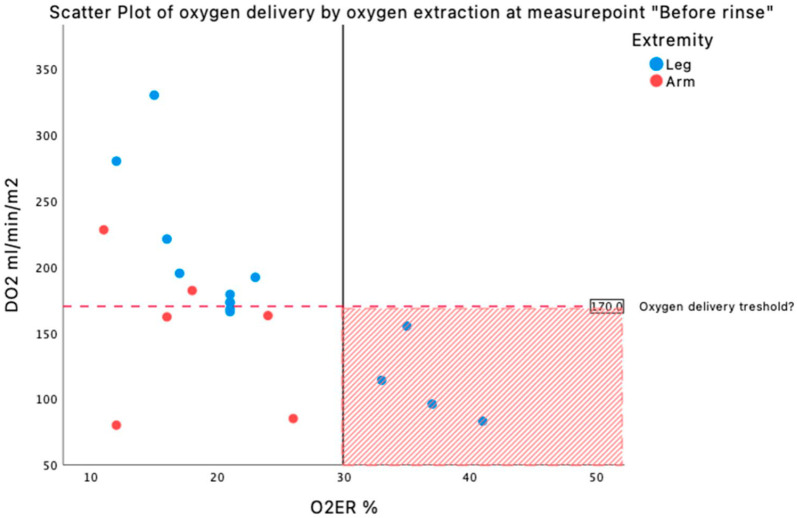
Scatter plot over oxygen delivery by oxygen extraction at the end of perfusion. Red marked area shows perfusions in this study which are above 30% of O_2_ER and below DO_2_ of 170 ml/min/m2, all legs.

When studying the parameters repeatedly for changes over time and for each extremity separately, there was a significant change in pO_2_, SaO_2_, hemoglobin, pump flowrate, CaO_2_, CvO_2_, DO_2_, O_2_ER, and SveO_2_ while VO_2_ remained unchanged. The pump flowrate decreased significantly during the perfusion in both extremities (*p*=<. 001) and hemoglobin decreased (legs *p*=<. 001, arms *p*=<. 01). This resulted in a significant decrease of DO_2_ over time (in legs from 209±65 to 180±66 ml/min/m^2^, *p*=<. 01 and in arms from 252±72 to 150±57 ml/min/m^2^, *p*=<. 01) (Table 3). O_2_ER increased significantly in arms during perfusion, with the highest value of 18% (*p*=. 03). The SveO_2_ increased and changed significantly during perfusion in legs (*p*=0.01), with the lowest value of 71±13% before initiating ECC (Table 3). The result showed that a DO_2_ above 170 ml/min/m_2_ kept the oxygen extraction below 30% for all observations (Figure 1).

**Table 3.** Characteristics during ILP. One way ANOVA repeated measure (“Before isolation” through “System circulation resumed”).

Parameters measured	Extremity	Afterinduction	Beforeisolation	ECC start	After chemo infusion	Before rinse	System circulation resumed	ANOVA*p*-Value
pO_2_ (kPa)	Leg	22±15	17±4	35±3***	33±3***	32±4***	18±5	<0.001***
	Arm	35±31	17±3	33±3**	29±6**	28±7*	17±3	0.001***
SaO_2_ (%)	Leg	98±2	98±1	99±1*	99±1***	99±1**	98±2	<0.01**
	Arm	98±1	98±1	98±1	99±2	99±1	98±1	0.26
Hb (g/L)	Leg	125±11	127±11	67±18***	64±16***	70±16***	117±12***	<0.001***
	Arm	113±8	111±7	74±9**	67±18**	68±20**	109±9	<0.01**
Lactate (mmol/L)	Leg	1.0±0.2	1.1±0.2	2.0±1.6*	2.6±1.1***	2.9±1.0***	1.8±0.6***	<0.001***
	Arm	1.0±0.3	1.1±0.3	5.0±0.7***	4.0±1.0**	3.9±1.6**	1.7±0.5*	<0.001***
Pump flow(L/min)	Leg	-	-	0.72±0.1	0.61±0.1**	0.58±0.2**	-	<0.001***
	Arm	-	-	0.36±0.1	0.26±0.1*	0.22±0.1*	-	<0.01**
CaO_2_ (mL/dL)	Leg	-	17±2	10±2***	10±2***	10±2***	16±2**	<0.001***
	Arm	-	15±1	11±2**	10±2**	10±3*	15±1	<0.01**
CvO_2_ (mL/dL)	Leg	-	12±3	8±2**	7±2**	8±2***	13±2	<0.001***
	Arm	-	13±1	9±1**	8±2*	8±2*	13±2	<0.01**
DO_2_ (mL/min/m^2^)	Leg	-	-	209±65	178±67**	180±66*	-	<0.01**
	Arm	-	-	252±72	174±62*	150±57**	-	<0.01**
VO_2_(mL/min/m^2^)	Leg	-	-	39±10	38±9	38±7	-	0.79
	Arm	-	-	33±6	28±9	26±10	-	0.12
O_2_ER (%)	Leg	-	30±14	20±7	24±10	24±9	22±12	0.21
	Arm	-	13±7	14±4*	16±6	18±6	11±6*	0.03*
SveO2 (%)	Leg	-	71±13	86±6**	82±10*	82±10*	80±12	0.01*
	Arm	-	88±6	90±4	89±6	86±6	89±5	0.30
NIRS treated (%)	Leg	65±13	64±13	72±11	73±11	75±11	72±12	0.21
	Arm	77±10	76±10	87±9	83±10	81±10	80±16	0.19
NIRS not treated (%)	Leg	66±14	67±14	67±12	68±13	68±14	66±11	0.12
	Arm	70±11	71±11	72±8	73±8	74±9	70±8	0.41

Blue highlighted fields represent measurements during ECC. Mean and standard deviation. Partial pressure of oxygen (PaO_2_), oxygen saturation (SaO_2_), hemoglobin (Hb), venous extremity oxygen saturation (SveO2), arterial oxygen content (CaO_2_), venous oxygen content (CvO_2_), oxygen delivery (DO_2_), oxygen consumption (VO_2_), oxygen extraction (O_2_ER), and Near infrared spectroscopy (NIRS). One-way ANOVA repeated measures (“Before isolation” through “System circulation resumed”) and a paired-sample T test for significant ANOVA parameters. *p*<0.05*, *p*<0.01**, *p*<0.001***

### Lactate measurements during ECC

Mean lactate on ECC was significantly higher in arms compared to legs (4.3±0.8 vs 2.51±1.1 mmol/L, *p*=0.01) (Table 2). Over time, lactate changed significantly during perfusion in both extremities (*p*=<. 001). In arms, lactate decreased during perfusion (5.0±0.7 at the start of ECC vs. 3.9±1.6 mmol/L at the end of ECC) but in legs lactate increased (2.0±1.6 mmol/L at the start of ECC vs. 2.9±1.0 mmol/L at the end of ECC). After perfusion, when system circulation was resumed, the lactate was 1.8±0.6 mmol/L in legs and 1.7±0.5 mmol/L in arms (Table 3).

### NIRS measurements during ECC

**Figure 2.**
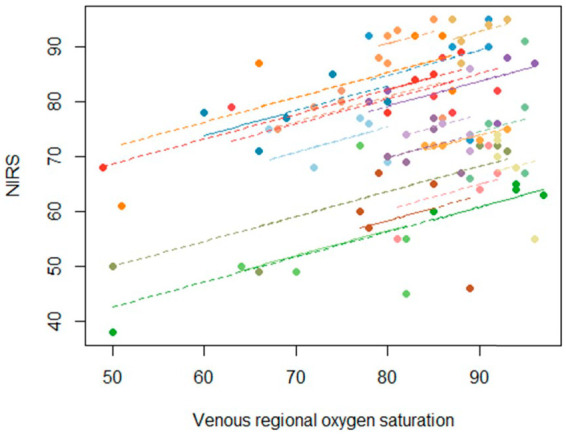
Repeated-measures correlation coefficient between SveO_2_ and rSO_2_ by NIRS. The repeated-measures correlation coefficients (r_rmCorr_), representing the strength of the linear association between SveO_2_ and NIRS. The inclinations between subjects are the same due to repeated measure total correlation coefficient 0.568 and length of the line represents the range for each individual three measures.

**Figure 3.**
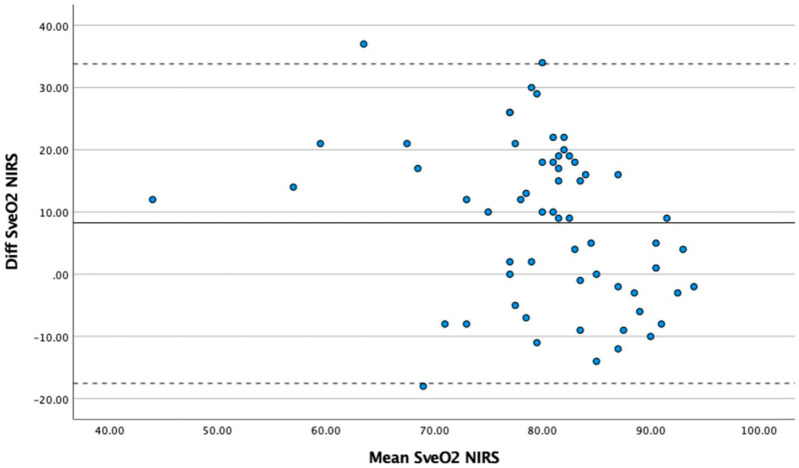
Bland–Altman plot. Agreement between SveO_2_ and rSO_2_ by NIRS. Bland–Altman plotting the agreement between venous regional oxygen saturation (SveO_2_) and near infrared spectroscopy (NIRS). All time points were included. Solid line indicates mean difference, and dotted lines indicate 95% limits of agreement.

Both arms and legs on ECC had higher NIRS values throughout the perfusion compared to the systemically perfused extremity. There was no significant change in NIRS values over time during ECC (in legs *p*=0.21 on ECC and *p*=0.12 on system circulation, in arms *p*=0.19 on ECC and *p*=0.41 on system circulation) (Table 3). Significant correlation could only be detected in the individuals’ measurements between the NIRS values and SveO_2_, r_rm_=0.568 (*p*<. 001, 95% CI 0.397–0.701) (Figure 2). There was no correlation in the individuals’ measurements between the NIRS values and PaO_2_, r_rm_ = 0.078 (*p*=0.49, 95% CI -0.145–0.294) and SaO_2_, r_rm_=0.165 (*p*=0.14, 95% CI -0.058–0.373). The Bland–Altman plot showed a mean of 80.13±9.0, a mean bias between SveO2 and NIRS was 8.26±13.0 and the, limit of agreement was −17.28–33.09, with an error of 32.5% between the two methods (Figure 3).

## DISCUSSION

In this study, the overall aim was to measure and describe oxygen metabolism in an isolated extremity during ILP, and to investigate whether rSO_2_ assessed by NIRS technique correlates and can be used interchangeable with invasive measurements of muscular oxygenation during ILP. The main finding was that DO_2_ above 170 ml/min/m^2^ kept the O_2_ER below 30% for all observations. Furthermore, a significant correlation was detected between rSO_2_ values and SveO_2_ with a moderate agreement between the two methods.

This is to our knowledge the first report on extremity-indexed oxygen supply/demand relationship in an isolated extremity during ILP. Perfusion research has to a great degree been focused on total body oxygen delivery with the aim to determine nadir values for preventing acute kidney injury after cardiac surgery.^8,10–15^ The results from this study however add an overview and certain comprehension of an extremity’s oxygen need, and provide the possibility for goal-directed delivery of sufficient amount of oxygen to the extremity tissue. Our results show that DO_2_ is nearly the same for both arms and legs during perfusion. The O_2_ER and VO_2_, however, are lower in arms compared to legs (Tables 2 and 3), which suggests that the metabolic need is lower in an arm than in a leg, probably due to different ratios between muscle/adipose tissue and bone. This was also shown in a study by Bevier et al.^39^ where correlation between muscle strength and VO_2_ max was found significant.^39^

Elevated lactate values may be an indication of inadequate oxygen delivery, a hypoperfusion, and a mismatch in the supply/demand relationship.^40^ Our result shows a higher level of lactate in arms compared to legs throughout the perfusion (Table 3). An explanation for this is most likely the addition of bank blood erythrocytes in the prime solution for arms, as bank blood contains a lactate level of around 8 mmol/L.^32^ During perfusion, the lactate value in arms decreases over time. The legs, however, have a significant rise in lactate during perfusion and might strengthen the above cogitation that legs require a higher amount of delivered oxygen per m^2^ than an arm to fulfill its metabolic need. If this relatively small lactate elevation during perfusion has any clinical impact on patients undergoing ILP is unknown. It is, however, known that when treating patients with isolated limb infusion (ILI), an established treatment similar to ILP but without perfusion and oxygenation, a progressive hypoxia, and acidosis is accepted.^41^ During perfusion, a significant decrease of pump flow in both extremities was seen. Obviously, this might be one explanation to the significant decrease of DO_2_ seen in arms and potentially also an explanation for the increased lactate values seen in legs during perfusion. This flow rate adjustment, often seen during ILP, is an important element of the procedure, where frequent alteration of the pump flow generates direct changes in the perfusion pressure of the extremity preventing leakage to and from the extremity, which is essential since leakage of chemotherapeutic agents from the extremity to the system circulation is potentially lethal for the patient.^42^ An attempt to keep DO_2_ at adequate levels despite a decreasing pump flow could be to elevate hematocrit and/or pO_2_, which to a certain extent can aid maintaining adequate DO_2_ levels and lactate levels.^10,12,14,33^

Even though DO_2_ of 170 ml/min/m^2^ in this study seems to be the lower DO_2_ limit to keep the O_2_ER at safe values (<30%), we found five patients (four arms and one leg) receiving a DO_2_ below 170 ml/min/m^2^ and keeping O_2_ER below 30%. Individual differences in tolerating lower range of DO_2_ values might be one explanation. O_2_ER over 30% was seen only in legs, as shown in Figure 1.

The study showed a statistically significant positive correlation between the NIRS values and SveO_2_ (r_rm_ = 0.568, *p* <. 001, CI 95% = 0.397–0.701). This finding supports former studies of the beneficial use of NIRS monitoring as a non-invasive way of measuring muscular oxygenation for detecting skeletal muscle ischemia.^27–31^ We measured NIRS values on both extremities during perfusion, which enabled us to compare oxygenation in the ECC perfused extremity to the extremity perfused by the system circulation. This guided us in what NIRS values not to go below to ensure a perfusion comparable to the patients’ native oximetry values in the systemically perfused extremity. The result shows that the ECC perfused extremities have higher NIRS values during perfusion compared to the systemic perfused extremity throughout the perfusion. Even though we found that NIRS was significantly correlated with SveO_2_, the Bland–Altman analysis showed a borderline agreement (error 32.5%) between the two methods.^37,38^ In this study, our acceptable error was set to 30% based on former studies.^37,38^ Our recommendation is therefore, considering the wide limit of agreement and an error of 32.5% that NIRS could be used as a complement to invasive measurements. Also, for comparing the ECC-perfused extremity against the extremity perfused by the systemic circulation, but not as a substitute for SveO_2_.

There are no vital organs in an isolated extremity, DO2 and VO2 should therefore be less than for total body oxygen consumption. But on the other hand, the temperature during ILP is approximately 40°C in the extremity. Oxygen affinity during these circumstances is decreased.^43^ This could be one explanation to the significant elevation of O_2_ER and lactate shown in legs in this study, even though NIRS values indicate that the extremities on ECC are better saturated than their own systemically provided extremity.

The main limitation of this study is the mix of prime solution between arms and legs. Another limitation is that our sample size is relatively small, but our measurements are within subjects and compared to initial values, which despite the small sample size and mix of prime solution make the findings valuable.

In summary, this study is to our knowledge the first study to measure and describe extremity indexed DO_2_, VO_2_, and O_2_ER in an isolated extremity during ILP. It seems that DO_2_ above 170 ml/min/m^2^ sufficiently supplies the extremity with its oxygen demand and keeps O_2_ER below 30%, which is below the considered upper safe limit for O_2_ER.^18^ Furthermore, invasive measurements of SveO_2_ cannot immediately be replaced by rSO_2_ values from the non-invasive NIRS technique, due to only moderate agreement between the two methods.
